# The longitudinal link between visual acuity and health-related quality of life in patients with diabetic retinopathy

**DOI:** 10.1186/1477-7525-6-95

**Published:** 2008-11-07

**Authors:** Louis S Matza, Matthew D Rousculp, Karen Malley, Kristina S Boye, Alan Oglesby

**Affiliations:** 1 Center for Health Outcomes Research, United BioSource Corporation, 7101 Wisconsin Avenue, Suite 600, Bethesda, MD 20814, USA; 2 Eli Lilly & Company, Indianapolis, IN, USA; 3 Malley Research Programming, Inc, Rockville, MD, USA; 4 Eli Lilly & Company, Indianapolis, IN, USA

## Abstract

**Background:**

This study characterized the degree of change in health-related quality of life (HRQL) associated with change in visual acuity among patients with diabetic retinopathy.

**Methods:**

Data are from a randomized, placebo-controlled trial of ruboxistaurin for vision loss in patients with diabetic retinopathy. Visual acuity was quantified as letters on the ETDRS visual acuity chart. HRQL was assessed with the 25-Item Visual Function Questionnaire (VFQ-25) and the SF-36. Patients were categorized into groups based on visual acuity change from baseline to month 18. HRQL change of these groups was compared using general linear models. Regression analyses examined visual acuity change defined continuously.

**Results:**

Patients (N = 535) were primarily Caucasian (81.9%) and male (64.1%); mean age = 59.3 years. Compared to patients whose visual acuity did not change, the group with > 10 letters vision loss had significantly greater decreases in all VFQ-25 subscales except ocular pain. SF-36 change scores did not correspond as closely to change in vision. Change in visual acuity defined continuously was significantly associated with change in all VFQ-25 scales except ocular pain (p < 0.0001).

**Conclusion:**

Change in visual acuity was associated with corresponding changes in HRQL among patients with diabetic retinopathy. Previous research has often defined vision loss as a loss of at least 15 letters on the ETDRS visual acuity chart. In the current study, however, a loss of at least 10 letters was associated with substantial declines in HRQL domains such as driving, dependency, role limitations, and mental health. These findings suggest that patients who experience vision loss of at least 10 letters may be appropriate targets of future research and clinical intervention.

## Background

Diabetic retinopathy is a retinal vascular disorder that develops to some degree in most patients with diabetes, leading to substantial vision loss for many patients [1-3]. Like other complications of diabetes, the risk and severity of this disorder can be reduced by controlling hyperglycemia and hypertension [4-8]. However, epidemiological research indicates that diabetic retinopathy remains common in patients with both type 1 and type 2 diabetes [9,10]. For example, a pooled analysis of age population-based high surveys estimated that 40.3% of adults 40 years and older with diabetes have retinopathy, and 8.2% of the population has vision-threatening retinopathy [3]. In qualitative studies involving focus groups and semi-structured interviews, patients have reported that diabetic retinopathy and the associated vision loss have a substantial impact on multiple domains of health-related quality of life (HRQL), including independence, mobility, leisure, and self-care activities [11,12].

Cross-sectional quantitative studies conducted at one point in time have also found that diabetic retinopathy is associated with impairment in functioning and overall HRQL [13-16]. Studies conducted in patients with diabetic retinopathy and other diabetes-related visual problems such as macular edema suggest that the degree of impact on HRQL is directly related to the reduction in visual acuity [13,17-19]. This inverse relationship between visual acuity and HRQL is consistent with results of research conducted among patients with a range of ocular diseases including macular degeneration, glaucoma, and cataract [13,17,20,21].

Although the association between visual acuity and HRQL at one point in time has been well established, little is known about this link over time. Thus, the purpose of the current study was to examine the degree of change in HRQL that is associated with visual acuity changes among patients with diabetic retinopathy. Patients were categorized into groups based on visual acuity changes during the first 18 months of a clinical trial, and these groups were compared with respect to change in both condition-specific and generic HRQL measures.

## Methods

### Data source

Data are from a 36-month, randomized, double-masked, placebo-controlled, parallel, multicenter trial of once-daily ruboxistaurin for vision loss in patients with diabetic retinopathy. The clinical findings and characteristics of this trial have been reported previously [22]. A total of 684 patients were randomized at 70 clinical sites in the United States. Randomized patients had type 1 or type 2 diabetes and HbA1c values ≤ 13%. To be eligible, patients had to have at least one eye that met ocular entry criteria: (a) Early Treatment Diabetic Retinopathy Study (ETDRS) retinopathy levels of > 47A and < 53E; (b) test-corrected visual acuity score of > 45 letters as measured by the ETDRS visual acuity protocol [23]; (c) no history of panretinal photocoagulation for diabetic retinopathy; (d) no evidence of glaucoma; and (e) no history of conditions affecting diabetes retinopathy progression. Ethical review boards provided written approval of the study protocol and the informed consent document. The study was initiated at each site after the principal investigator and the study sponsor obtained approval documents. The principal investigator at each site was responsible for obtaining informed consent from each patient or legal representative and for obtaining the appropriate signatures on the informed consent document prior to performing any procedures or administering any study drug.

### Measures

#### National Eye Institute Visual Function Questionnaire – 25-item version (VFQ-25)

This 25-item patient-reported questionnaire is a short form of a 51-item survey designed to assess the influence of visual impairment on health-related quality of life (HRQL). The content of the original questionnaire was derived from multi-condition focus groups [24]. The 25-item version has been shown to have adequate reliability and validity in a sample consisting of participants with age-related cataracts, age-related macular degeneration, diabetic retinopathy, primary open-angle glaucoma, or cytomegalovirus retinitis [25]. The instrument has also been shown to be strongly associated with vision, independent of severity of retinopathy and other complications associated with type 1 diabetes [19]. In previous studies, the VFQ-25 has been able to detect functional impairment associated with a wide range of ocular conditions [20,25-29].

The VFQ-25 yields a total score and 11 visual subscale scores: general vision, ocular pain, near vision, distance vision, social function, mental health, role limitations, dependency, driving, color vision, and peripheral vision. Each subscale score ranges from 0 to 100, with higher scores representing better HRQL. The instrument also includes a general health item. In this trial, the general health item was completed only if patients did not complete the SF-36, and consequently, results of this item are not reported in the current paper. Consistent with the developers' instructions and previous publications, the total score was computed without including the general health item.

#### Medical Outcomes Study (MOS) 36-Item Short Form Health Survey (SF-36)

The SF-36 is a 36-item measure used to gather information about the patient's perceived health. The 36 items are completed by the patient and gather information about eight health concepts: physical functioning, physical role functioning, bodily pain, general health, vitality, social functioning, emotional role functioning, and mental health. Higher scores on the SF-36 indicate that the patient has reported better-perceived health [30,31].

### Measure of visual acuity

Visual acuity was determined as part of an ophthalmologic examination (including slit-lamp biomicroscopy, intraocular pressure, and ophthalmoloscopy) at screening and each study visit. Visual acuity was quantified in terms of number of letters as measured by the Early Treatment Diabetic Retinopathy Study (ETDRS) visual acuity protocol [23]. These scores have a possible range of 0 to 100 letters, with higher scores indicating better visual acuity. For the current analysis, visual acuity was defined at each time point (i.e., baseline and month 18) as each individual's ETDRS rating for his/her better eye (referred to as "best eye ETDRS"), regardless of which eye is used at other time points, and regardless of whether the best eye met criteria for being included as a "study eye." Previous research has found that binocular acuity can be inferred from measures of monocular acuity in the better eye, particularly when assessing functional impairment associated with visual acuity, and separate assessment of binocular acuity is not required [32].

### Statistical analyses

The health outcomes measures used in the current analysis were administered at baseline, month 18, and month 36. The current analysis focused on change from baseline to month 18 because of the sample size limitations associated with health outcomes data at month 36. Baseline analyses, including descriptive statistics, were performed using the sample of all enrolled patients. Analyses of change in visual acuity and/or HRQL were performed with the subset of patients who had at least one VFQ-25 subscale present at both baseline and month 18 (this subset is called the "change analysis sample"). Analyses were conducted to assess whether patients meeting this criterion differed from patients who were excluded. The primary measure of HRQL in the study was the condition-specific VFQ-25. Secondary analyses examined group differences in the generic SF-36.

Descriptive analyses were conducted to summarize demographic/clinical variables, visual acuity (ETDRS), and HRQL. The scoring of all questionnaires followed the guidelines set forth by the instrument developers. Categorical variables were summarized in terms of frequencies and percentages, and continuous variables were summarized in terms of means and standard deviations. Preliminary analyses were conducted to examine reliability and validity of the VFQ-25 to ensure that this instrument performed adequately in patients with diabetic retinopathy. Cronbach's alpha for each VFQ-25 scale was computed to assess internal consistency reliability. Construct validity of the VFQ-25 was examined with Spearman correlations between the VFQ-25 scales and visual acuity at baseline and month 18. In this study, correlations were interpreted as small (0.10), moderate (0.30), or large (0.50) following the guidelines proposed by Cohen [33].

The primary analyses focused on the link between visual acuity (i.e., ETDRS) and HRQL (i.e., the VFQ-25 and to a lesser extent, the SF-36) over time. This link was analyzed with change in visual acuity defined both categorically and continuously. First, categorical analyses were conducted to characterize the HRQL decreases/gains associated with various degrees of change in visual acuity. Patients were categorized into five groups based on ETDRS change from baseline to month 18: Improved by ≥ 10 letters, Improved by 5 to 9 letters, No change (-4 to +4 letters), Worsened by 5 to 9 letters, and Worsened by ≥ 10 letters. A series of 12 general linear models (GLMs) with Scheffe's post hoc pairwise comparisons were conducted to compare the VFQ-25 change scores of the five groups, when controlling for age, gender, and baseline visual acuity. In these models, the change group is a five-level independent variable. Age and baseline visual acuity are continuous covariates, and gender is a categorical covariate. The dependent variables were change scores from baseline to month 18. A parallel set of 12 models was conducted with the SF-36 subscales as the dependent variables.

To examine the relationship between the VFQ-25 subscales and visual acuity defined as a continuous variable, 12 additional GLMs were conducted. These models are the same as those described above, except visual acuity was entered as a continuous variable rather than a five-level categorical variable.

Finally, as an exploratory descriptive analysis, change scores for each VFQ-25 item were calculated for each of the five ETDRS change groups. These scores are presented to provide a rough indication of the precise areas of HRQL and functioning that may tend to change along with visual acuity in patients with diabetic retinopathy.

SAS statistical software version 8.2 was used for all analyses. All statistical tests were two-tailed and conducted with significance level for testing fixed at 0.05.

## Results

### Sample characteristics

Baseline demographic and clinical characteristics for the total sample (N = 684), change analysis sample (N = 535), and patients excluded from the change analysis sample (N = 149) are presented in Table 1. There were no statistically significant differences between the change analysis sample and the excluded sample in age, gender, type of diabetes, duration of diabetes, body mass index (BMI), or baseline ETDRS. The only difference between the two samples was in ethnicity, as the change analysis sample had a somewhat larger percentage of Caucasian patients. The change analysis sample was primarily male (64.1%) and Caucasian (81.9%), with a mean age of 59.3 years. This sample was primarily diagnosed with type 2 diabetes (88.0%), and the sample had a mean BMI of 32.8 at baseline.

**Table 1:** Baseline demographics and clinical characteristics

**Characteristic**	Change analysis sample* (N = 535)	**Excluded (N = 149)**	**Total sample (N = 684)**	**p-value^†^**
Age (mean years, SD)	59.3 ± 10.8	59.3 ± 10.8	59.3 ± 10.8	0.97
Gender (n, %)				
Male	343 (64.1%)	90 (60.4%)	433 (63.3%)	0.44
Female	192 (35.9%)	59 (39.6%)	251 (36.7%)	
Ethnicity (n, %)				
Caucasian	438 (81.9%)	94 (63.1%)	532 (77.8%)	<0.0001
African descent	42 (7.9%)	28 (18.8%)	70 (10.2%)	
East/Southeast Asian	21 (3.9%)	6 (4.0%)	27 (3.9%)	
Hispanic	17 (3.2%)	20 (13.4%)	37 (5.4%)	
Other	17 (3.2%)	1 (0.7%)	18 (2.6%)	
Type of diabetes (n, %)				
Type 1	64 (12.0%)	16 (10.7%)	80 (11.7%)	0.77
Type 2	471 (88.0%)	133 (89.3%)	604 (88.3%)	
Duration of diabetes (years; mean ± SD)	16.3 ± 8.3	15.1 ± 7.5	16.0 ± 8.1	0.11
Body mass index (kg/m^2^; mean ± SD)	32.8 ± 6.9	32.8 ± 9.1	32.8 ± 7.4	0.95
ETDRS visual acuity rating (letters; mean ± SD)				
Best eye	81.3 ± 8.2	80.1 ± 8.6	81.1 ± 8.3	0.12
Worst eye	72.2 ± 15.0	69.3 ± 17.2	71.6 ± 15.6	0.06
Right eye	77.5 ± 12.2	75.6 ± 12.0	77.1 ± 12.2	0.08
Left eye	76.0 ± 13.6	73.9 ± 16.8	75.6 ± 14.3	0.15

*Patients with visual acuity assessment and at least one VFQ-25 subscale at both baseline and month 18

^†^Continuous variables compared with t-tests; 2-level categorical variables such as gender compared with Fisher exact test; categorical variables with more than two levels compared with chi-square analyses

ETDRS = Early Treatment Diabetic Retinopathy Study; SD = standard deviation; VFQ-25 = 25-Item Visual Function Questionnaire

### VFQ-25 and ETDRS descriptive statistics

Baseline descriptive statistics for the VFQ-25 are presented in Table 2. Mean subscale scores ranged from 69.5 (general vision) to 94.6 (color vision), and the total score was 84.1. At baseline, there was substantial heterogeneity in the sample, with scores ranging from 0 to 100 in several subscales. The multi-item subscales of the VFQ-25 generally had adequate internal consistency reliability. Cronbach's alpha was 0.68 for the ocular pain subscale, 0.64 for the social function subscale, and at least 0.72 for all other subscales. Correlations with ETDRS visual acuity rating were statistically significant (p < 0.01) for all subscales except ocular pain. The correlation coefficients were in the moderate range (i.e., ≥ 0.30) for the total score and the general vision, near vision, distance vision, role limitations, and driving subscales. In the change analysis sample, mean best eye ETDRS visual acuity was 81.3 letters at baseline (Table 1) and 81.4 letters at month 18.

**Table 2:** Distributional characteristics, internal consistency reliability, and construct validity of VFQ-25 scales at baseline

**Scale**	**N**	**Mean**	**SD**	**Range**	**Number of items**	**Cronbach's alpha^†^**	**Spearman correlations with ETDRS**
General vision	670	69.5	17.4	20.0 – 100.0			0.39***
Ocular pain	671	89.1	15.2	12.5 – 100.0	2	0.68	0.02
Near vision	671	75.8	21.7	8.3 – 100.0	3	0.81	0.38***
Distance vision	671	82.9	17.9	16.7 – 100.0	3	0.72	0.35***
Social function	671	93.9	12.9	12.5 – 100.0	2	0.64	0.25***
Mental health	671	77.2	21.3	0.0 – 100.0	4	0.79	0.28***
Role limitations	670	81.4	23.1	0.0 – 100.0	2	0.75	0.32***
Dependency	670	91.3	17.4	0.0 – 100.0	3	0.83	0.29***
Driving	632	81.1	20.7	0.0 – 100.0	3	0.73	0.34***
Color vision	665	94.6	14.7	0.0 – 100.0			0.12**
Peripheral vision	669	88.4	19.4	25.0 – 100.0			0.25***
Overall VFQ-25 score	671	84.1	13.5	16.2 – 100.0	25	0.93	0.40***

^†^Computed only for multi-item scales

*p < 0.05; **p < 0.01; ***p < 0.001

ETDRS = Early Treatment Diabetic Retinopathy Study Visual Acuity Rating; SD = standard deviation; VFQ-25 = 25-Item Visual Function Questionnaire

The five change groups that are examined in the subsequent analyses were compared in terms of age and baseline visual acuity. There were no significant differences among the groups in age. However, there were some group differences in baseline visual acuity. Baseline ETDRS visual acuity ratings for the five groups were as follows: 72.2, Improved by ≥ 10 letters; 79.0, Improved by 5 to 9 letters; 83.0, No change (-4 to +4 letters); 81.5, Worsened by 5 to 9 letters, and 79.9, Worsened by ≥ 10 letters. The group that improved by ≥ 10 letters had significantly worse baseline visual acuity than the other four groups (p < 0.01). The only other significant difference was between the group that improved by 5 to 9 letters and the no change group (p < 0.01).

### The association between visual acuity and HRQL

Results of GLMs examining the link between best eye ETDRS visual acuity and the VFQ-25 are presented in Table 3. Change in visual acuity was generally associated with corresponding changes in most VFQ-25 scores. However, pairwise comparisons revealed no statistically significant differences in mean VFQ-25 change scores among the two improvement groups and the no change group. Compared with the improved by ≥ 10 letters group, the worsened by 5 to 9 letters group had significantly different change scores in the VFQ-25 dependency subscale and total score. The group that worsened by ≥ 10 letters had significantly different change scores than most of the other groups, including the no change group, in all VFQ-25 scales except ocular pain. There were no differences among groups in the ocular pain.

**Table 3:** ANCOVAs comparing VFQ-25 change scores among groups of patients differing in visual acuity change from baseline to month 18†

**Change in** ** VFQ-25 score** ** mean (SD)**	**Improved ** **by ≥ 10 ** **(N = 32–35)**	**Improved ** **by 5 to 9 ** **(N = 80–85)**	**No change** ** (-4 to +4)** ** (N = 300–323)**	**Worsened** ** by 5 to 9 ** **(N = 47–53)**	**Worsened** ** by ≥ 10 ** **(N = 36–39)**	**Overall** ** F value**	**Change in** ** best visual** ** acuity** ** p-value**	**Significant** **pairwise** **comparisons**
General vision	9.1 (17.0)	3.3 (16.9)	-0.2 (15.8)	-1.5 (14.1)	-13.3 (19.1)	5.98***	<0.0001	D***, G***, I***, J*
Ocular pain	1.8 (15.2)	-0.1 (16.3)	2.2 (14.8)	2.4 (13.9)	1.6 (15.0)	0.68	0.74	
Near vision	6.3 (17.7)	3.6 (17.9)	1.1 (17.2)	-4.5 (16.1)	-15.8 (24.9)	6.16***	<0.0001	D***, G***, I***
Distance vision	2.7 (12.8)	1.4 (14.8)	0.3 (14.3)	-5.6 (14.9)	-16.2 (19.5)	8.45***	<0.0001	D***, G***, I***, J*
Social function	2.9 (7.5)	-0.9 (9.9)	-0.5 (10.6)	-3.3 (14.3)	-11.2 (24.1)	5.24***	<0.0001	D***, G***, I***
Mental health	7.3 (17.9)	2.3 (15.1)	0.5 (16.9)	-3.3 (22.9)	-17.5 (28.1)	6.39***	<0.0001	D***, G***, I***, J*
Role limitations	3.6 (15.6)	3.8 (21.0)	0.2 (17.9)	-6.4 (23.6)	-21.5 (31.4)	7.59***	<0.0001	D***, G***, I***, J*
Dependency	3.3 (14.0)	-3.0 (13.8)	-0.3 (14.3)	-7.5 (22.8)	-26.1 (32.8)	13.35***	<0.0001	C*, D***, G***, I***, J***
Driving	0.1 (18.9)	-2.1 (14.1)	-1.8 (15.4)	-6.9 (19.1)	-22.9 (35.1)	7.91***	<0.0001	D***, G***, I***, J**
Color vision	2.3 (15.8)	-1.5 (14.7)	0.6 (12.4)	-3.8 (17.4)	-11.5 (31.3)	3.56***	0.0001	D*, G*, I***
Peripheral vision	5.7 (20.2)	-2.6 (17.3)	-0.5 (18.6)	-5.0 (22.0)	-16.7 (32.1)	4.52***	<0.0001	D***, G*, I***
Overall VFQ-25 score	3.9 (9.1)	0.2 (10.1)	0.1 (8.9)	-4.4 (11.4)	-15.6 (19.2)	13.99***	<0.0001	C**, D***, G***, I***, J***

Pairwise comparisons:

A: Improved by ± 10 vs. Improved by 5 to 9; B: Improved by ± 10 vs. No change (-4 to +4);

C: Improved by ± 10 vs. Worsened by 5 to 9; D: Improved by ± 10 vs. Worsened by ± 10;

E: Improved by 5 to 9 vs. No change (-4 to +4); F: Improved by 5 to 9 vs. Worsened by 5 to 9;

G: Improved by 5 to 9 vs. Worsened by ± 10; H: No change (-4 to +4) vs. Worsened by 5 to 9;

I: No change (-4 to +4) vs. Worsened by ± 10; J: Worsened by 5 to 9 vs. Worsened by ± 10

^†^Covariates: age in years, gender, best eye visual acuity at baseline

*p < 0.05; **p < 0.01; ***p < 0.001

ANCOVAs = analysis of covariance; SD = standard deviation; VFQ-25 = 25-Item Visual Function Questionnaire

A parallel set of models was conducted to assess whether the five change groups differ in HRQL change as assessed by SF-36 subscales (Table 4). Results suggest that change in the SF-36 did not correspond as closely as the VFQ-25 to change in visual acuity. For example, the SF-36 reflected slight declines in overall quality of life from baseline to month 18, even among the groups that improved in visual acuity. The only statistically significant pairwise comparison between groups was between the no change group and the worsened by ≥ 10 letters group in the mental health subscale.

**Table 4:** ANCOVAs comparing SF-36 change scores among groups of patients differing in visual acuity change from baseline to month 18†

**Change in** ** SF-36 ** **score mean (SD)**	**Improved** ** by ≥ 10** ** (N = 35)**	**Improved** ** by 5 to 9** ** (N = 85)**	**No change** ** (-4 to +4)** ** (N = 323)**	**Worsened** ** by 5 to 9 ** **(N = 53)**	**Worsened** ** by ≥ 10** ** (N = 39)**	**Overall** ** F value**	**Change in** ** best visual** ** acuity** ** p-value**	**Significant** ** pairwise** ** comparisons**
Physical functioning	-4.0 (15.1)	-4.5 (21.5)	-2.9 (19.7)	-5.1 (17.9)	-5.7 (25.7)	1.88	0.96	
Role-physical	-10.7 (36.5)	-3.5 (41.4)	-9.2 (38.4)	-6.6 (46.0)	-21.2 (43.5)	2.14*	0.23	
Role-emotional	-6.7 (19.5)	-6.7 (30.8)	1.1 (25.2)	-3.1 (26.4)	-2.6 (30.0)	1.31	0.20	
Pain index	-2.0 (23.0)	-2.9 (25.5)	-2.6 (24.7)	-9.0 (22.5)	-9.5 (29.0)	1.15	0.37	
Mental health	-1.5 (16.3)	-1.5 (18.1)	0.4 (14.4)	-0.8 (12.2)	-8.4 (15.7)	2.67*	0.04	I*
Social functioning	-5.0 (23.9)	-1.8 (22.8)	-3.1 (20.5)	-4.2 (15.5)	-9.0 (26.1)	2.04*	0.58	
Vitality	-4.7 (23.3)	-4.2 (21.2)	-2.7 (20.1)	-3.6 (17.0)	-6.5 (20.9)	2.26*	0.95	
General health perceptions	1.3 (13.3)	-2.0 (21.1)	-1.6 (15.5)	-2.3 (15.5)	-8.3 (16.5)	3.32**	0.11	
Physical component summary	-1.4 (7.7)	-1.3 (10.0)	-2.2 (8.7)	-2.9 (8.8)	-4.5 (11.7)	2.31*	0.50	
Mental component summary	-1.5 (9.2)	-1.2 (9.5)	0.3 (7.2)	-0.4 (7.4)	-2.6 (8.9)	1.85	0.35	

Pairwise comparisons:

I: No change (-4 to +4) vs. Worsened by ± 10

^†^Covariates: age in years, gender, best eye visual acuity at baseline

*p < 0.05; **p < 0.01; ***p < 0.001

ANCOVAs = analysis of covariance; SD = standard deviation

In addition, 12 GLMs were conducted to examine the relationship between change in visual acuity defined as a continuous variable and change in the VFQ-25 scales, controlling for age, gender, and visual acuity at baseline. In all models, except the one with the VFQ-25 ocular pain subscale as the dependent variable, change in visual acuity was significantly associated with change in the VFQ-25 scale (p < 0.0001). Baseline visual acuity was also associated with the following four dependent variables: Distance Activities, Dependency, Driving, and the VFQ-25 total score (p < 0.05). Age and gender were not significantly associated with the VFQ-25 scales in any of the models.

### Comparing change in individual VFQ-25 items among the five ETDRS change groups

Mean change in each individual item of the VFQ-25 was computed for each of the five groups from baseline to month 18 (Table 5). In general, most individual items tended to reflect improvement among the groups that improved in ETDRS and worsening among the groups that decline in ETDRS. Items that followed this logical pattern included (item 2) present eyesight rating, (item 5) difficulty reading newsprint, (item 6) seeing well up close, (item 7) difficulty finding objects on a crowded shelf, (item 8) difficulty reading street signs, (item 17) accomplish less due to vision, (item 18) work less due to vision, and (item 21) frustrated due to vision.

**Table 5:** Mean change in individual items of the VFQ-25 for five groups of patients categorized by ETDRS change

**Baseline to month 18 change in each item of the VFQ-25^† ^mean (SD)**	**Five groups of patients categorized by ETDRS change from baseline to month 18**				
	
	**Improved by ≥ 10 (N = 28–35)**	**Improved by 5 to 9 (N = 78–85)**	**No change (-4 to +4) (N = 286–323)**	**Worsened by 5 to 9 (N = 45–53)**	**Worsened by ≥ 10 (N = 27–39)**
General vision (Item 2)	9.1 (17.0)	3.3 (16.9)	-0.2 (15.8)	-1.5 (14.1)	-13.3 (19.1)
Ocular pain					
4. Amount pain	2.9 (24.1)	1.8 (22.8)	3.3 (19.5)	2.4 (19.2)	5.1 (20.0)
19. Amount time: pain	0.7 (14.2)	-2.1 (16.0)	1.2 (16.1)	2.4 (17.2)	-1.9 (18.5)
Near vision					
5. Reading normal newsprint	9.6 (24.6)	5.5 (24.5)	1.0 (23.9)	-1.4 (25.9)	-14.5 (34.7)
6. Seeing well up close	8.6 (25.7)	4.3 (25.1)	1.9 (22.7)	-7.7 (20.1)	-12.8 (26.8)
7. Finding objects on crowded shelf	2.2 (21.6)	0.9 (19.6)	0.3 (20.8)	-3.3 (20.8)	-19.9 (31.0)
Distance vision					
8. Reading street signs	5.7 (24.3)	3.3 (22.2)	-0.9 (20.1)	-4.2 (23.4)	-16.0 (29.0)
9. Going down stairs at night	-0.8 (14.6)	0.0 (22.3)	0.6 (21.3)	-5.2 (24.2)	-10.9 (24.9)
14. Going out to movies/plays	3.3 (10.9)	-0.3 (16.7)	1.1 (15.8)	-7.8 (23.7)	-20.8 (27.1)
Social function					
11. Seeing how people react	2.3 (9.6)	-0.6 (14.5)	-0.5 (15.3)	-6.7 (23.8)	-13.5 (32.4)
13. Visiting others	3.7 (10.9)	-0.6 (8.7)	-0.5 (12.1)	-1.0 (14.0)	-9.0 (25.3)
Mental health					
3. Amount true: worry	6.4 (21.3)	9.4 (22.2)	4.7 (26.3)	0.0 (25.9)	-8.3 (28.3)
21. Amount true: frustrated	12.9 (33.4)	0.9 (25.7)	0.3 (27.7)	-3.4 (41.7)	-17.3 (37.7)
22. Amount true: no control	7.9 (30.8)	-0.6 (27.3)	-1.8 (26.1)	-6.7 (34.0)	-21.8 (37.7)
25. Amount true: embarrassment	2.1 (15.3)	-0.6 (18.9)	-1.1 (19.1)	-2.9 (27.4)	-22.4 (39.2)
Role limitations					
17. Accomplish less	5.0 (27.7)	3.8 (25.7)	-0.9 (22.8)	-6.6 (26.9)	-23.1 (34.1)
18. Limited in endurance	2.1 (15.3)	3.8 (23.6)	1.3 (20.9)	-6.1 (32.5)	-19.9 (35.9)
Dependency					
20. Stay home most of time	5.7 (15.0)	-2.6 (14.4)	-1.0 (13.8)	-5.3 (18.7)	-21.8 (31.5)
23. Rely too much on others' word	2.9 (18.0)	-3.2 (18.0)	0.3 (20.8)	-8.2 (32.0)	-28.2 (39.0)
24. Need help from others	1.4 (21.0)	-3.2 (17.2)	-0.2 (19.9)	-9.1 (32.1)	-28.2 (40.2)
Driving					
15C. Daylight familiar places	-0.8 (10.0)	1.6 (12.2)	-1.7 (14.6)	-4.8 (17.8)	-25.0 (38.7)
16. Nighttime familiar places	-0.9 (25.9)	-5.7 (25.6)	-0.8 (19.2)	-6.1 (30.7)	-7.1 (34.6)
16A. Difficult conditions	-4.3 (24.2)	-2.9 (20.9)	-0.9 (20.5)	-8.7 (21.9)	-13.0 (32.1)
Color vision					
12. Difficulty matching clothes	2.3 (15.8)	-1.5 (14.7)	0.6 (12.4)	-3.8 (17.4)	-11.5 (31.3)
Peripheral vision					
10. Seeing objects off to side	5.7 (20.2)	-2.6 (17.3)	-0.5 (18.6)	-5.0 (22.0)	-16.7 (32.1)

^†^Three items of the VFQ-25 are not included in this table: 1 (general health), 15a (never driven/given up driving), and 15b (reason for giving up driving). Item 1 was not completed by most participants in this study. Items 15a and 15b follow skip-patterns and are not rated on the same scale as the other items.

ETDRS = Early Treatment Diabetic Retinopathy Study; SD = standard deviation; VFQ-25 = 25-Item Visual Function Questionnaire

## Conclusion

Change in visual acuity was associated with change in multiple domains of HRQL during this 18-month trial. Categorical analyses suggested that patients with a loss of 10 letters on the ETDRS visual acuity chart (i.e., a two-line loss) had significantly greater mean declines in nearly all VFQ-25 scales than any other group of patients. Among the other four visual acuity change groups, HRQL change scores also followed logical patterns. For example, the group with the greatest visual acuity improvement tended to have the greatest gains in VFQ-25 subscale scores. However, differences in VFQ-25 scores among these four groups were relatively small and mostly not statistically significant.

Previous research has often defined vision loss as a loss of at least 15 letters, which is the equivalent of three lines on the ETDRS visual acuity chart [8,23]. Current results suggest that a lower threshold may be used to define meaningful vision loss. For example, a loss of at least 10 letters was associated with substantial declines in key HRQL domains such as driving, dependency, role limitations, and mental health. These results are consistent with a previous study indicating that even mild to moderate visual impairment has a notable impact on psychological functioning [34]. Based on current results, patients who experience vision loss of at least 10 letters may be appropriate targets of future research and clinical intervention.

This study also provides additional psychometric support for the VFQ-25. Previously, this measure has been validated in samples combining patients with a range of eye conditions including cataracts, macular degeneration, diabetic retinopathy, glaucoma, or low vision from any cause [25,35]. In the current sample which consists entirely of patients with diabetic retinopathy, all multi-item scales demonstrated adequate internal consistency reliability. In addition, all scales except ocular pain and color vision demonstrated construct validity through significant correlations in the moderate to large range with best eye visual acuity. The two subscales with weaker correlations assessed constructs that are not directly impaired by diabetic retinopathy and were not expected to be significantly related to visual acuity. The VFQ-25 also demonstrated responsiveness to change in visual acuity, particularly among patients with vision loss of at least 10 letters. In comparison to this condition-specific instrument, the generic SF-36 seemed relatively unresponsive to change, possibly because the SF-36 assesses a wide range of characteristics that are not directly related to visual acuity. This distinction between the two instruments is consistent with previous research indicating that condition-specific patient-reported outcome measures tend to be more responsive to change than generic measures [36-38]. In sum, the reliability, validity, and responsiveness demonstrated in this study support the use of the VFQ-25 for assessing HRQL among patients with diabetic retinopathy, particularly in studies examining change over time. Generic measures such as the SF-36 have other strengths. For example, generic measures such as the SF-36 can be used to make comparisons to the general population, estimate the relative impact of various medical conditions, and derive a utility value summarizing health status for cost-effectiveness modeling [39-44]. Given the different strengths of condition-specific and generic measures, the choice of a patient-reported measure for any individual study should take into account the study design, sample characteristics, hypotheses, and aims.

Analyses were somewhat limited by the sample size. For example, the group of 39 patients whose visual acuity deteriorated by at least 10 letters is heterogeneous, with visual acuity loss ranging from 10 to 52 letters (one patient lost 52 letters, another lost 39 letters, and the other 37 patients lost between 10 and 27 letters). Analyses comparing subgroups of patients with different levels of vision loss within this group of patients could help identify whether there is a threshold beyond which HRQL is affected. However, the current sample size is not large enough to support division of these 39 patients into subgroups. Another limitation of this study is that sufficient HRQL data are only available at two points in time (i.e., baseline and 18 months). Although the clinical trial did extend to 36 months, there were not enough data at this third time point to justify further analysis. Thus, the current results do not provide insight into the ways visual acuity and HRQL may change over time. These changes may be gradual, but it is also possible that there is a point in the process of visual acuity loss when most patients begin to experience functional changes as well. Future research with larger samples and assessments at multiple time points is needed to better understand the link between visual acuity and HRQL over time.

Despite these limitations, the current study provides strong initial support for the hypothesis that visual acuity loss is associated with a corresponding decline in HRQL among patients with diabetic retinopathy. Importantly, the findings suggest that visual acuity loss of at least 10 letters is likely to have a significant impact on functioning. Thus, in clinical settings, patients who decline to this moderate degree should be questioned about functional changes. Patients whose visual acuity loss has in fact begun to affect their HRQL may benefit from early intervention aimed at bolstering the affected functional domains.

## Abbreviations

ANCOVA: analysis of covariance; BMI: body mass index; ETDRS: Early Treatment Diabetic Retinopathy Study; GLM: general linear model; HRQL: health-related quality of life; SD: standard deviation; SF-36: Medical Outcomes Study (MOS) 36-Item Short Form Health Survey; VFQ-25: 25-Item Visual Function Questionnaire

## Competing interests

LM works for UBC, a company that received funds from Eli Lilly & Company for this research. KB is an employee of Eli Lilly & Company. MR and AO were employees of Eli Lilly & Company at the time this research was conducted. KM is a subcontractor who received payment from UBC for the time she spent working on this project.

## Authors' contributions

LM co-directed this study and was the primary writer of the manuscript. He played a key role in hypothesis generation, study design, statistical analysis, and data interpretation. MR co-directed this study. He played a key role in hypothesis generation, study design, statistical analysis, data interpretation, and manuscript editing. KB and AO assisted with hypothesis generation, study design, data interpretation, and editing of the manuscript. KM performed the statistical programming for this study.
